# List of the names of the referees for the studies that were published in the São Paulo Medical Journal/Evidence for Health Care during the year 2008.

**DOI:** 10.1590/S1516-31802008000600012

**Published:** 2008-11-06

1. **Agnaldo Pereira Cedenho** — Universidade Federal de São Paulo (Unifesp).
2. **Alcides Augusto Salzedas Netto** — Universidade Federal de São Paulo (Unifesp)
3. **Amílcar Martins Giron** — Faculdade de Medicina da Universidade de São Paulo (FMUSP).
4. **Ana Beatriz Alvarez Perez** — Universidade Federal de São Paulo (Unifesp).
5. **Ana Maria Schiefer** — Universidade Federal de São Paulo (Unifesp).
6. **André Luis Alves de Lemos** — Universidade Federal de São Paulo (Unifesp).
7. **Antonio Hélio Oliani** — Faculdade de Medicina de São José do Rio Preto (Famerp).
8. **Antonio José Gonçalves** — Faculdade de Ciências Médicas da Santa Casa de São Paulo (FCMSCSP).
9. **Aylton Figueira Junior** — Universidade Municipal de São Caetano do Sul (USCS).
10. **Aytan Miranda Sipahi** — Faculdade de Medicina da Universidade de São Paulo (FMUSP).
11. **Caio Parente Barbosa** — Fundação e Faculdade de Medicina do ABC (FMABC).
12. **Carlos Alberto Pires Pereira** — Universidade Federal de São Paulo (Unifesp).
13. **Carmita Helena Najjar Abdo** — Faculdade de Medicina da Universidade de São Paulo (FMUSP).
14. **Claudio Torres de Miranda** — Universidade Federal de Alagoas (UFAL).
15. **Cristina Muccioli** — Universidade Federal de São Paulo (Unifesp).
16. **Daniel Deheinzelin** — Faculdade de Medicina da Universidade de São Paulo (FMUSP).
17. **Daniella Márcia Bahia Kerbauy** — Universidade Federal de São Paulo (Unifesp).
18. **Dirce Maria Trevisan Zanetta** — Faculdade de Saúde Pública (FSP), Universidade de São Paulo (USP).
19. **Domingo Marcolino Braile** — Faculdade de Medicina de São José do Rio Preto (Famerp).
20. **Durval Rosa Borges** — Universidade Federal de São Paulo (Unifesp).
21. **Edina Mariko Koga da Silva** — Universidade Federal de São Paulo (Unifesp).
22. **Edmund Chada Baracat** — Faculdade de Medicina da Universidade de São Paulo (FMUSP).
23. **Eduardo Alexandrino Servolo de Medeiros** — Universidade Federal de São Paulo (Unifesp).
24. **Eduardo da Frota Carrera** — Universidade Federal de São Paulo (Unifesp).
25. **Elcio dos Santos Oliveira Vianna** — Faculdade de Medicina de Ribeirão Preto, Universidade de São Paulo (FMRP-USP).
26. **Emmanuel de Almeida Burdmann** — Faculdade de Medicina de São José do Rio Preto (Famerp).
27. **Fernando Antonio de Almeida** — Pontifícia Universidade Católica de São Paulo (PUC-SP).
28. **Fernando Ferreira Costa** — Universidade Estadual de Campinas (Unicamp).
29. **Flávio Faloppa** — Universidade Federal de São Paulo (Unifesp).
30. **Francisco Aparecido Belfort** — Universidade Federal de São Paulo (Unifesp).
31. **Hakaru Tadokoro** — Universidade Federal de São Paulo (Unifesp).
32. **Heráclito Barbosa de Carvalho** — Faculdade de Medicina da Universidade de São Paulo (FMUSP).
33. **Humberto Saconato** — Universidade Federal do Rio Grande do Norte (UFRN).
34. **Isabela Martins Benseñor** — Faculdade de Medicina da Universidade de São Paulo (FMUSP).
35. **João Hamilton Romaldini** — Pontifícia Universidade Católica de Campinas (PUC-Campinas).
36. **João Victor Salvajoli** — Hospital A. C. Camargo, Fundação Antonio Prudente.
37. **José Antonio Rocha Gontijo** — Universidade Estadual de Campinas (Unicamp).
38. **José Carlos Costa Baptista-Silva** — Universidade Federal de São Paulo (Unifesp).
39. **José Mauro da Silva Rodrigues** — Pontifícia Universidade Católica de São Paulo (PUC-SP).
40. **José Roberto Lapa e Silva** — Universidade Federal do Rio de Janeiro (UFRJ).
41. **Julio César Rodrigues Pereira** — Faculdade de Saúde Pública (FSP), Universidade de São Paulo (USP).
42. **Julisa Chamorro Lascasas Ribalta** — Universidade Federal de São Paulo (Unifesp).
43. **Katia Ferreira Güenaga** — Universidade Metropolitana de Santos (Unimes).
44. **Laércio Joel Franco** — Faculdade de Medicina de Ribeirão Preto, Universidade de São Paulo (FMRP-USP).
45. **Luiz Antônio Nogueira-Martins** — Universidade Federal de São Paulo (Unifesp).
46. **Luiz Eugênio Garcez Leme** — Faculdade de Medicina da Universidade de São Paulo (FMUSP).
47. **Luiz Sérgio Fonseca de Azevedo** — Faculdade de Medicina da Universidade de São Paulo (FMUSP).
48. **Marcelo Luis Abramides Torres** — Faculdade de Medicina da Universidade de São Paulo (FMUSP).
49. **Maria de Lourdes Lopes Chauffaille** — Universidade Federal de São Paulo (Unifesp).
50. **Maria do Patrocínio Tenório Nunes** — Faculdade de Medicina da Universidade de São Paulo (FMUSP).
51. **Maria Lúcia de Pinho-Apezzato** — Faculdade de Medicina da Universidade de São Paulo (FMUSP).
52. **Marilza Vieira Cunha Rudge** — Faculdade de Medicina de Botucatu, Universidade Estadual Paulista (FMB/Unesp).
53. **Mauricio Mota de Avelar Alchorne** — Universidade Federal de São Paulo (Unifesp).
54. **Maurício Simões Abrão** — Faculdade de Medicina da Universidade de São Paulo (FMUSP).
55. **Mauro Roberto Tucci** — Faculdade de Medicina da Universidade de São Paulo (FMUSP).
56. **Milton de Arruda Martins** — Faculdade de Medicina da Universidade de São Paulo (FMUSP).
57. **Moacir Fernandes de Godoy** — Faculdade de Medicina de São José do Rio Preto (Famerp).
58. **Olavo Pires de Camargo** — Faculdade de Medicina da Universidade de São Paulo (FMUSP).
59. **Orlando César de Oliveira Barretto** — Faculdade de Medicina da Universidade de São Paulo (FMUSP).
60. **Paola Zucchi** — Universidade Federal de São Paulo (Unifesp).
61. **Paulo Henrique Waib** — Faculdade de Medicina de Marília (Famema).
62. **Rachel Riera** — Universidade Federal de São Paulo (Unifesp).
63. **Rita de Cássia Barradas Barata** — Faculdade de Ciências Médicas da Santa Casa de São Paulo (FCMSCSP).
64. **Rosa Maria Rodrigues Pereira** — Faculdade de Medicina da Universidade de São Paulo (FMUSP).
65. **Rosana Fiorini Puccini** — Universidade Federal de São Paulo (Unifesp).
66. **Ruth Guinsburg** — Universidade Federal de São Paulo (Unifesp).
67. **Sérgio Aron Ajzen** — Universidade Federal de São Paulo (Unifesp).
68. **Sérgio Mancini Nicolau** — Universidade Federal de São Paulo (Unifesp).
69. **Silvana Maria Quintana** — Faculdade de Medicina de Ribeirão Preto, Universidade de São Paulo (FMRP-USP).
70. **Suzana Angélica Silva Lustosa** — Universidade Federal de São Paulo (Unifesp).
71. **Vilmon de Freitas** — Universidade Federal de São Paulo (Unifesp).
72. **Virgínia Fernandes Moça Trevisani** — Universidade Federal de São Paulo (Unifesp).
73. **Walter José Gomes** — Universidade Federal de São Paulo (Unifesp).
74. **Wilson Roberto Oliveira Milani** — Universidade Federal de São Paulo (Unifesp).
75. **Wladimir Taborda** — Universidade Federal de São Paulo (Unifesp).

